# Sex Is Associated with the Success or Failure of Manipulation Alone for Joint Stiffness Associated with Rotator Cuff Repair

**DOI:** 10.3390/jcm11237192

**Published:** 2022-12-03

**Authors:** Kohei Yamaura, Yutaka Mifune, Atsuyuki Inui, Hanako Nishimoto, Shintaro Mukohara, Tomoya Yoshikawa, Issei Shinohara, Tatsuo Kato, Takahiro Furukawa, Yuichi Hoshino, Takehiko Matsushita, Ryosuke Kuroda

**Affiliations:** Department of Orthopedic Surgery, Graduate School of Medicine, Kobe University, Kobe 650-0017, Japan

**Keywords:** shoulder stiffness, rotator cuff tear, manipulation, arthroscopic capsular release

## Abstract

Purpose: One-stage arthroscopic rotator cuff repair with manipulation has been recently performed for rotator cuff tears with shoulder stiffness, whereas some patients require capsular release due to severe stiffness that is difficult to treat with manipulation. The purpose of this study was to analyze patient backgrounds and related factors of success or failure of manipulation alone for the treatment of shoulder stiffness associated with rotator cuff tears. Methods: This study included 64 patients with rotator cuff tears and shoulder stiffness who underwent arthroscopic rotator cuff repair with manipulation alone or with manipulation and capsular release of the glenohumeral joint at our institution between January 2015 and September 2019. The patients were divided into two groups: those whose shoulder stiffness could be improved by manipulation alone (Manipulation group) and those whose stiffness could not be improved by manipulation alone and required capsular release (Capsular release addition group). Analysis was performed between the two groups regarding patient backgrounds and related factors, including rotator cuff tear morphology and range of motions pre- and postoperatively. Results: Exactly 45 patients and 19 patients were included in Manipulation group and Capsular release addition group, respectively. A comparison between the two groups showed that patient age (*p* = 0.0040), sex (*p* = 0.0005), and injury due to trauma (*p* = 0.0018) were significantly related to the success or failure of manipulation alone. Multivariate logistic regression analysis on these three factors showed that sex (odds ratio, 5.5; *p* = 0.048) was significantly associated with the success or failure of manipulation alone. In both groups, the passive ROM of all patients improved at the last postoperative follow-up compared to their pre-operative values (*p* < 0.001), except for internal rotation in the Capsular release addition group (*p* = 0.49). Conclusion: Young male patients who have shoulder stiffness associated with rotator cuff tears should be considered for arthroscopic capsular release rather than manipulation.

## 1. Introduction

Rotator cuff tears are a common shoulder joint disorder that affects approximately 10% of the population over the age of 60 [1], causing many functional disabilities in daily life, such as limited range of motion (ROM), pain, and muscle weakness [2]. Active ROM of the shoulder joint is usually limited with rotator cuff tears, and passive ROM is often preserved; however, rotator cuff tears with shoulder stiffness that limit passive ROM are often encountered clinically [3], with a complication rate of approximately 11–20% [4,5,6,7]. When considering mild passive ROM restriction, this complication rate increases to approximately 40% [8]. The pathogenesis of shoulder stiffness following rotator cuff tears has been shown to involve pain secondary to inflammation of the bursa and synovium [9], disuse of the joint, secondary muscle weakness [10], and soft tissue contractures around the glenohumeral joint [11].

The difficulty in treatment of rotator cuff tears with shoulder stiffness is due to the paradoxical nature of postoperative immobilization for tendon–bone interface healing after rotator cuff repair and ROM exercises for stiffness improvement [8]. The acquisition of ROM was conventionally considered to be preferable prior to the intervention of rotator cuff tear repair [12,13]. However, the delay in rotator cuff repair until the recovery of ROM may lead to complications, such as rotator cuff tear deterioration, progressive muscle atrophy, and fatty degeneration [14,15], which are poor prognostic factors for retears after rotator cuff repair [16,17,18]. Recently, more reports have demonstrated the efficacy of one-stage rotator cuff repair and manipulation [19] or one-stage rotator cuff repair and capsular release [20,21]. Manipulation is generally performed first under general anesthesia, as in our institution, whereas capsular release is only added when passive ROM requires further improvement [20].

To the best of our knowledge, although there are several comparative studies on the postoperative clinical outcomes of manipulation alone and manipulation with capsular release [22,23], there are no reports that have examined patient backgrounds and related factors that determine whether manipulation alone is sufficient or if capsular release is necessary due to too much stiffness. Therefore, this study aimed to analyze patient backgrounds and related factors of success or failure of manipulation alone for the treatment of shoulder stiffness associated with rotator cuff tears.

## 2. Materials and Methods

### 2.1. Participant Selection

This study was approved by the institutional review board. This study included 64 patients with rotator cuff tears and shoulder stiffness who underwent arthroscopic rotator cuff repair with manipulation alone or with manipulation and capsular release of the glenohumeral joint at our institution from January 2015 to September 2019. The inclusion criteria were as follows: (1) full-thickness rotator cuff tear confirmed by magnetic resonance imaging (MRI) by the senior author (Y.M.), who is a shoulder arthroscopic surgeon; (2) passive ROM limitation under general anesthesia (<135° forward flexion, <60° external rotation) [5,12]; (3) arthroscopic rotator cuff repair and manipulation or capsular release of the glenohumeral joint in addition to manipulation; and (4) follow-up for at least 12 months post-operatively. The exclusion criteria were as follows: (1) irreparable rotator cuff tear, (2) previous fracture or surgery of the ipsilateral shoulder joint, and (3) progressive osteoarthritis.

### 2.2. Study Design

This was a retrospective case-control study using the background and related factors of patients with rotator cuff tears and shoulder stiffness who underwent arthroscopic rotator cuff repair or capsular release of the glenohumeral joint in addition to manipulation. In this study, the patients were divided into two groups: those whose shoulder stiffness could be improved by manipulation alone (Manipulation group) and those whose stiffness could not be improved by manipulation alone and required capsular release (Capsular release addition group). Factors that may affect shoulder stiffness associated with rotator cuff tears were evaluated, including patient background, such as sex, age, medical history, dominant side, presence of traumatic injury, duration of symptoms, number and involvement of torn tendons, tear size, and passive ROM pre-operatively and at the last follow-up postoperatively. A multivariate analysis was also performed on the factors that showed significant differences in the two-group comparison to identify statistically significant factors.

### 2.3. Assessment of Related Factors

The aforementioned patient background data were collected from medical records, with the exception of the number and involvement of torn tendons and tear size, which were based on intraoperative arthroscopic findings. Tears were categorized as small (<1 cm), medium (1–3 cm), large (3–5 cm), and massive (>5 cm) [24]. The involvement of torn tendons was only applied to full-thickness tears, which were categorized as the following: type 1, only supraspinatus involved; type 2, supraspinatus and subscapularis involved together; type 3, supraspinatus and infraspinatus involved together; and type 4, all three tendons involved [25]. For pre- and postoperative passive ROM measurements, forward flexion, abduction, external rotation, and internal rotation were assessed with the patient in a sitting position in the outpatient clinic. External rotation was measured with the arm at the side and the elbow flexed at 90°. Internal rotation was measured at the vertebral level that the patient could reach with the tip of the thumb, which was converted into numbers for statistical analysis as follows: thoracic vertebrae 1 to 12 levels were expressed as 1 to 12, lumbar vertebrae 1 to 5 levels were expressed as 13 to 17, and the sacrum or hip was expressed as 18 [26,27]. Lastly, ROM was measured independently by a senior author (Y.M.), who is a shoulder arthroscopic surgeon.

### 2.4. Surgical Procedure

All surgeries were performed by the senior author (Y.M.), who is a shoulder arthroscopic surgeon, with the patient in a beach chair position under general anesthesia. After securing the patient in the surgical position, ROM under general anesthesia was measured for forward flexion, abduction, and external rotation of the affected shoulder joint. All 64 patients with ROM limitation underwent manipulation prior to rotator cuff repair.

### 2.5. Manipulation and Arthroscopic Capsular Release under General Anesthesia

The manipulation technique was performed in a similar manner to that of the previous report [28]. First, the anterior joint capsule was released by external rotation with the upper arm at the side of the trunk. When the joint capsule was released, the characteristic crepitus sound and markedly improved ROM were observed [19]. Next, the anterior inferior joint capsule was released by external rotation with the shoulder joint abducted at 90°, and the lower joint capsule was released by forward flexion. Subsequently, the posterior inferior joint capsule was released by internal rotation with the shoulder joint abducted at 90°, and the posterior joint capsule was released by internal rotation of the shoulder joint in horizontal adduction. Finally, the anterior superior and superior joint capsules were released by internal and external rotations with the shoulder joint in extension. After manipulation, passive ROM was measured again. If the patient met the requirements (≥170° forward flexion, ≥170° abduction, and ≥70° external rotation), the patient would undergo rotator cuff repair. If not, an additional capsular release would be performed prior to rotator cuff repair. Posterior and anterior portals were set up for arthroscopic examination, and anterior–inferior capsular release was performed in all patients. The rotator interval and the middle and inferior glenohumeral ligaments were released using an electrocautery probe from the anterior portal during posterior arthroscopy. After confirming improvement of passive ROM, rotator cuff repair was performed.

### 2.6. Rotator Cuff Repair

Five portals were used in all surgeries: the anterior, anterolateral, posterolateral, posterior, and anchor portals. The arthroscopy field of view was secured using a shaver and an electrocautery probe, and the rotator cuff tear was probed to identify the torn tendon and confirm the tear size. For supraspinatus (SSP) and infraspinatus (ISP) suturing, the suture bridge method with two medial anchors and two lateral anchors was used. The single-row or suture bridge method with one medial and one lateral anchor was also used depending on the tear size and tear configuration. Furthermore, the subscapularis (SSC) tendon repair was performed using the single-row method with full-thickness tears over the upper 25% of the attachment [7].

### 2.7. Post-Operative Rehabilitation

For post-operative therapy, all patients wore abduction slings for 6 weeks post-operatively, and both groups were treated with the same rehabilitation program. Active ROM exercises of the elbow, wrist, and fingers were allowed during the early postoperative period. For exercises without the use of abduction slings, passive ROM exercises were allowed from 3 weeks post-operatively, whereas active ROM exercises were allowed from 6 weeks post-operatively. Muscle strengthening exercises were performed from 12 weeks post-operatively, and return to manual labor was allowed at 3 months post-operatively for light loads and at 6 months postoperatively for heavy loads.

### 2.8. Statistical Analysis

Statistical analyses were performed using the SPSS software (PASW, version 18.0, SPSS Inc., Chicago, IL, USA) and R version 2.7.1 (The R Foundation for Statistical Computing Platform, Vienna, Austria), and data were expressed as means ± standard deviation. Sex, dominant side of the affected joint, medical history, presence of traumatic injury, and involvement of the torn rotator cuff between the two groups were analyzed using the χ2 test, and the size of the rotator cuff tears was analyzed using the Mann–Whitney test. Pre- and post-operative ROM comparisons within each group were analyzed using a paired *t*-test, whereas comparisons of other factors between the two groups were performed using an unpaired *t*-test. Factors that showed a significant difference between the two groups were examined using multivariate analysis. Statistical significance was set at *p* < 0.05. The number of sample sizes in the a priori analysis was not calculated because there were no previous studies of two-group comparisons similar to the present study. Therefore, the strategy was to perform a post-hoc power analysis for the main parameters to evaluate whether the sample size was sufficient in this study.

## 3. Results

### 3.1. Study Group

Of the 64 patients who underwent arthroscopic rotator cuff repair or capsular release of the glenohumeral joint in addition to manipulation, 45 were classified into Manipulation group, and 19 were classified into Capsular release addition group. Regarding surgical complications, there was only one case of glenoid fracture due to manipulation in Manipulation group. The fractured glenoid fragment was removed since it was extremely small and did not cause shoulder instability. No other surgical complications occurred in either group.

### 3.2. Patient Demographics

A comparison of patient backgrounds between the two groups is shown in Table 1. While the Capsular release addition group was significantly younger (*p* = 0.0050) and comprised more males (*p* = 0.0044) than Manipulation group, there were no significant differences in the dominant side of the affected joint, duration of symptoms, or the post-operative follow-up period between the two groups. Regarding the mechanism of injury, 21 patients (46.7%) were due to trauma and 24 patients (53.3%) were not due to obvious trauma in Manipulation group, whereas 17 patients (89.5%) were due to trauma and two patients (10.5%) were not due to obvious trauma in Capsular release addition group. This showed that the Capsular release addition group had significantly more injuries due to trauma than the Manipulation group (*p* = 0.0018). Regarding medical history, four patients (8.9%) had diabetes mellitus, two patients (4.4%) had Parkinson’s disease, and five patients (11.1%) had hypertension in Manipulation group. Meanwhile, four patients (21.1%) had diabetes mellitus, no patients had Parkinson’s disease, and one patient (5.3%) had hypertension in Capsular release addition group. There were no significant differences in specific diseases between the two groups.

### 3.3. Morphology of the Rotator Cuff Tear (Number and Type of Torn Tendons, Tear Size)

A comparison of the intraoperative arthroscopic findings between the two groups is shown in Table 2. The mean number of torn rotator cuff tendons was 1.6 ± 0.7 tendons in the Manipulation group and 1.7 ± 0.6 tendons in the Capsular release addition group, showing no significant difference in the number of rotator cuff tears between the two. The involvement of torn rotator cuff tendons in the Manipulation group were as follows: 24 patients (53.3%) with type 1 (SSP), five patients (11.1%) with type 2 (SSP + SSC), 11 patients (24.4%) with type 3 (SSP + ISP), and five patients (11.1%) with type 4 (SSP + ISP + SSC). On the other hand, the involvement of torn rotator cuff tendons in the Capsular release addition group was as follows: seven patients (36.8%) with type 1 (SSP), six patients (31.6%) with type 2 (SSP + SSC), four patients (21.1%) with type 3 (SSP + ISP), and two patients (10.5%) with type 4 (SSP + ISP + SSC). There was no significant difference in the involvement of torn rotator cuff tendons between the two groups. The rotator cuff tear sizes in the Manipulation group and the Capsular release addition group were small tears in three (6.7%) and two (10.5%) patients, medium tears in 40 (88.9%) and 13 (68.4%) patients, large tears in two (4.4%) and four (21.1%) patients, and massive tears in 0 (0%) and 0 (0%) patients, respectively. Similar to the previous findings, there was no significant difference in the size of rotator cuff tears between the two groups.

### 3.4. ROM

The comparison of ROM between the two groups preoperatively and at the last follow-up postoperatively is shown in Table 3. There were no significant differences between the two groups in forward flexion, abduction, external rotation, and internal rotation pre- and postoperatively.

The comparison of ROM within each group preoperatively and at the last follow-up post operatively is shown in Table 4. In both groups, the passive ROM of all patients improved at the last postoperative follow-up compared to their pre-operative values (*p* < 0.001) except for internal rotation in the Capsular release addition group (*p* = 0.49).

### 3.5. Multivariate Analysis of Related Factors between the Two Groups

A multivariate logistic analysis was performed to evaluate significant factors, including age, sex, and traumatic injury, regarding the feasibility of manipulation alone under general anesthesia for the treatment of shoulder stiffness associated with rotator cuff tears. The results of the multivariate logistic analysis are shown in Table 5. Sex (odds ratio [OR], 5.5; *p* = 0.048) was significantly related to success or failure of manipulation alone, whereas age (OR, 0.93; *p* = 0.060) and injury due to trauma (OR, 4.8; *p* = 0.078) were not significantly related to the success or failure of manipulation alone.

## 4. Discussion

The greatest strength of this study is that it is the first report to evaluate patient backgrounds and factors related to the success or failure of manipulation alone under general anesthesia for shoulder stiffness associated with rotator cuff tears. To the best of our knowledge, while there are several reports on the clinical outcomes of one-stage arthroscopic rotator cuff repair combined with manipulation and/or capsular release for this condition [22,23], there are no reports that have evaluated the predictors of success or failure of manipulation alone. The results of the two-group comparison in this study showed that male patients who have shoulder stiffness associated with rotator cuff tears are difficult to treat with manipulation alone. In other words, capsular release rather than manipulation may be indicated, especially for male patients who present with shoulder stiffness associated with rotator cuff tears. Regarding the involvement of frozen shoulder and medical history, although diabetes mellitus [29], hypertension [30], subarachnoid hemorrhage [31], and Parkinson’s disease [32] have been suggested to be involved, medical history showed no significant involvement in the success or failure of manipulation alone in this study. Similarly, regarding torn tendon morphology or tear size, no significant association was noted with the success or failure of manipulation alone. The results of the present study also indicated that shoulder stiffness associated with rotator cuff tears may be due to the same pathology as primary frozen shoulder, rather than the effects on the joint capsule characteristics of rotator cuff tears.

Shoulder stiffness associated with rotator cuff tears is commonly encountered in clinical practice, with a reported incidence of approximately 11–20% [4,5,6,7]. While the complication rate of restricted shoulder ROM after arthroscopic rotator cuff repair has been reported to be approximately 4.3–4.9% [8,33], and risk factors have been suggested to include age, single tendon rotator cuff repair, partial articular-sided supraspinatus tendon avulsion (PASTA) repair, and workers’ compensation [11,33], there are very few reports investigating the pathogenesis and risk factors of shoulder stiffness associated with rotator cuff tears [34]. Understanding the pathogenesis and related factors of this condition is important since pre-operative concomitant ROM restriction has been shown to influence post-operative ROM restriction and quality of life [21,33].

The difficulty in treating shoulder stiffness associated with rotator cuff tears is due to the paradox of treating rotator cuff tears and joint contractures [8]. Recently, the efficacy of one-stage treatment in managing this condition has been reported [20,21]. A systematic review in 2017 showed that one-stage surgery for rotator cuff tears with shoulder stiffness had similar post-operative clinical outcomes to surgery for rotator cuff tears without shoulder contractures [8]. Notably, there are two main methods of treatment for shoulder stiffness: manipulation or arthroscopic capsular release. There are many reports on the efficacy of manipulation under ultrasound-guided cervical nerve root block for frozen shoulder [28,35,36], which is now established as a beneficial treatment method that can be performed as an outpatient surgery. On the other hand, arthroscopic capsular release was reported to have significantly improved postoperative pain relief and ROM [37,38] Despite these studies, the superiority between these two methods remains debatable. There have been cases in which sufficient ROM cannot be achieved using manipulation alone. Therefore, in this study, manipulation was first performed under general anesthesia, followed by rotator cuff repair. Patients who could not obtain sufficient ROM by manipulation alone then underwent arthroscopic capsular release. Of the 64 patients with shoulder contractures associated with rotator cuff tears, 19 (30%) required additional capsular release. McGrath et al. [20] first performed capsular release in addition to manipulation under general anesthesia for shoulder stiffness associated with rotator cuff tears in patients who had difficulty achieving adequate ROM. Although their definition of stiffness differed in terms of a more severe pre-operative ROM restriction, additional capsular release was required in 17 of their 25 cases.

The manipulation technique in this study was the same as in previous reports [28,35,36], starting with the anterior joint capsule tear by external rotation and gradually widening the torn part of the joint capsule. Although complications of manipulation rarely occur, they include rotator cuff tears, humeral fractures, shoulder joint dislocations, traction nerve injuries, and glenoid fractures [39,40]. Takahashi et al. reported that one of 68 patients developed an avulsion fracture of the inferior glenoid rim, which was similar in the present study as one of 56 patients developed an avulsion fracture of the anterior inferior glenoid [28]. Regarding the arthroscopic capsular release technique, it remains debatable whether the release of the joint capsule should be circumferential or partial. In the latest 2021 systematic review including 811 shoulders (629 patients) from 18 eligible articles, the outcomes of three techniques—anterior–inferior capsular release, anterior–inferior–posterior capsular release, and 360-degree capsular release—were investigated [41]. The results suggested that anterior–inferior capsular release, which has a less extensive release, may improve function and pain. In the present study, the anterior–inferior capsular release was performed in all cases, and no complications were observed. This is consistent with the technique as arthroscopic capsular release is a safe procedure with a low incidence of complications, including axillary nerve injury.

The results of this study demonstrate two interesting points. First, approximately 70% of patients with shoulder stiffness associated with rotator cuff tears can improve ROM with general anesthesia manipulation alone prior to arthroscopy. Acquisition of favorable ROM by manipulation prior to arthroscopy may facilitate arthroscopic procedures and contribute to shorter operative time due to the ease of manipulation and reduced risk of axillary nerve injury in arthroscopic capsular release [42,43]. Second, approximately 30% of patients with shoulder stiffness associated with rotator cuff tears, especially male patients, have difficulty improving ROM by manipulation alone and require arthroscopic capsular release. To prevent complications such as fractures due to aggressive manipulation, it is clinically meaningful to understand the factors that make it difficult to treat with manipulation. Therefore, male patients with shoulder stiffness associated with rotator cuff tears should be carefully treated with pathetic manipulation, and if favorable ROM is difficult to acquire, arthroscopic capsular release should be performed without hesitation.

Despite these findings, this study has several limitations. First, the number of patients, particularly those who could not be treated with manipulation alone, was relatively small. Since this study is the first to examine the success or failure of manipulation alone for shoulder stiffness associated with rotator cuff tears, there are no similar previous studies. The post-hoc power analysis of the χ2 analysis for sex and trauma injury and the two-group comparison for age were high, at 0.86, 0.91, and 0.92, respectively, suggesting that the small sample size had little effect on the results. Second, there was no objective standard for the forward flexion, abduction, and external rotation angles applied by manipulation. To the best of our knowledge, although there are many reports on the application of manipulation and capsular release for shoulder stiffness, there are no definitive standardized criteria. Finally, the study did not assess pre- and postoperative clinical outcomes using clinical scores, and the follow-up period was only 1 year postoperatively. Considering these points, further studies with a larger number of patients and longer follow-up should evaluate the factors associated with these two groups, including the clinical scores.

## 5. Conclusions

Young male patients who have shoulder stiffness associated with rotator cuff tears should be considered for arthroscopic capsular release rather than manipulation.

## Figures and Tables

**Table 1:** Comparison of patient demographics between two groups.

	Manipulation Group	Capsular Release Addition Group	*p* Value
Number of patients	45	19	-
Mean age at surgery ^α^ (y)	65.9 ± 9.9	56.8 ± 5.8	0.00050 *
Sex ^β^ (Number (%))	Male: 23 (51.1)	Male: 17 (89.5)	0.0044 *
	Female: 22 (48.9)	Female: 2 (10.5)	
Number of dominant or nondominant side of affected shoulder ^β^ (Number (%))	Dominant side: 19	Dominant side: 10	0.58
	Nondominant side: 26	Nondominant side: 9	
Mean duration of symptom ^α^ (months)	11.2 ± 10.2	7.2 ± 3.9	0.10
Mean postoperative follow-up period ^α^ (months)	21.9 ± 9.2	18.2 ± 5.8	0.11
Medical history ^β^ (Number (%))	Diabetes: 4 (8.9)	Diabetes: 4 (21.1)	0.20
	Parkinson’s disease: 2 (4.4)	Parkinson’s disease: 0 (0)	0.99
	Hypertension: 5 (11.1)	Hypertension: 1 (5.3)	0.66
Injury mechanism ^β^ (Number (%))	Trauma: 21 (46.7)	Trauma: 17 (89.5)	0.0018 *

Results are reported as means ± SD. * Significant difference (*p* < 0.05) between groups. ^α^: Comparison between groups using unpaired *t*-test. ^β^: Comparison between groups using χ2 analysis.

**Table 2:** Comparison of the intraoperative arthroscopic findings between the two groups.

	Manipulation Group (*n* = 45)	Capsular Release Addition Group (*n* = 19)	*p* Value
Mean number of torn tendons ^α^ (Number)	1.6 ± 0.7	1.7 ± 0.7	0.40
Involvement of torn tendons ^β^ (Number (%))	Type 1: 24 (53.3) (SSP)	Type 1: 7 (36.8) (SSP)	0.25
	Type 2: 5 (11.1) (SSP + SSC)	Type 2: 6 (31.6) (SSP + SSC)	
	Type 3: 11 (24.4) (SSP + ISP)	Type 3: 4 (21.1) (SSP + ISP)	
	Type 4: 5 (11.1) (SSP + ISP + SSC)	Type 4: 2 (10.5) (SSP + ISP + SSC)	
Tear size ^γ^ (Number (%))	small: 3 (6.7)	small: 2 (10.5)	0.24
	medium: 40 (88.9)	medium: 13 (68.4)	
	large: 2 (4.4)	large: 4 (21.1)	
	massive: 0 (0)	massive: 0 (0)	

Results are reported as means ± SD. ^α^: Comparison between two groups using the unpaired *t*-test. ^β^: Comparisons between the two groups using χ2 analysis. ^γ^: Comparisons between groups using the Mann–Whitney test. SSP, supraspinatus; ISP, infraspinatus; SSC, subscapularis.

**Table 3:** Comparison of passive ROM before and after surgery between the two groups.

		Manipulation Group (*n* = 45)	Capsular Release Addition Group (*n* = 19)	*p* Value
Before surgery	Forward flexion (degrees)	106.4 ± 22.3	113.0 ± 22.6	0.30
	Abduction (degrees)	84.0 ± 22.3	93.2 ± 21.6	0.14
	External rotation (degrees)	44.0 ± 15.4	42.1 ± 18.4	0.67
	Internal rotation ^α^	15.2 ± 3.3	14.9 ± 3.6	0.74
At the last follow-up after surgery	Forward flexion (degrees)	171.8 ± 10.5	172.6 ± 7.3	0.75
	Abduction (degrees)	164.0 ± 13.9	161.6 ± 13.0	0.52
	External rotation (degrees)	73.1 ± 10.8	72.1 ± 15.1	0.76
	Internal rotation ^α^	12.6 ± 2.2	12.9 ± 1.9	0.61

Results are reported as means ± SD. Internal rotation ^α^: thoracic vertebrae levels 1–12 were expressed as 1–12, lumbar vertebrae levels 1–5 were expressed as 13–17, and the sacrum or hip was expressed as 18.

**Table 4:** Comparison of passive ROM within each group before and after surgery.

		before Surgery	At the Last Follow-Up after Surgery	*p* Value
Manipulation group (*n* = 45)	Forward flexion (degrees)	106.4 ± 22.3	171.8 ± 10.5	<0.001 *
	Abduction (degrees)	84.0 ± 22.3	164.0 ± 13.9	<0.001 *
	External rotation (degrees)	44.0 ± 15.4	73.1 ± 10.8	<0.001 *
	Internal rotation ^α^	15.2 ± 3.3	12.6 ± 2.2	<0.001 *
Capsular release addition group (*n* = 19)	Forward flexion (degrees)	113.0 ± 22.6	172.6 ± 7.3	<0.001 *
	Abduction (degrees)	93.2 ± 21.6	161.6 ± 13.0	<0.001 *
	External rotation (degrees)	42.1 ± 18.4	72.1 ± 15.1	<0.001 *
	Internal rotation ^α^	14.9 ± 3.6	12.9 ± 1.9	0.49

Results are reported as means ± SD. * Significant difference (*p* < 0.05) between groups. Internal rotation ^α^: thoracic vertebrae levels 1–12 were expressed as 1–12, lumbar vertebrae levels 1–5 were expressed as 13–17, and the sacrum or hip was expressed as 18.

**Table 5:** Results of the multivariate logistic analysis.

	Odds Ratio (95% CI)	*p* Value
Age	0.93 (0.86–1.0)	0.060
Sex	5.5 (1.0–30)	0.048 *
Trauma injury	4.8 (0.85–27)	0.077

* Significant difference (*p* < 0.05).

## Data Availability

The data presented in this study are available on request from the corresponding author. The data are not publicly available due to confidentiality concerns.
